# Lifestyle Factors and Diet-Disease-Related Knowledge: A Network Psychometric Analysis of Cardiovascular Health Literacy Among Lebanese Adults

**DOI:** 10.3390/nu18132196

**Published:** 2026-07-06

**Authors:** Elite A. Dib, Sofi G. Julien

**Affiliations:** Department of Nutrition and Food Sciences, Faculty of Arts and Sciences, Holy Spirit University of Kaslik, Jounieh P.O. Box 446, Lebanon; elite.a.dib@net.usek.edu.lb

**Keywords:** nutritional knowledge, Mediterranean diet, EBICglasso estimator, biomarker awareness, cardiovascular biomarkers, health literacy, Lebanon, cross-sectional study, node centrality indices

## Abstract

Background/Objectives: Diet-disease-related knowledge (DDRK) is theorized to foster healthier lifestyles; however, the structural interconnectedness between nutritional knowledge and personal biomarker awareness is not well understood. This study aimed to identify independent predictors of DDRK and cardiovascular health (CVH) literacy among Lebanese adults using a psychometric framework. Methods: A cross-sectional convenience-sampled online survey was conducted among 406 Lebanese adults. Standard validated questionnaires were used, including the GNKQ-Section 4, GPAQ, and MEDAS. CVH literacy was computed as the total awareness of five main CV biomarkers: total cholesterol, LDL, HDL, triglycerides, and HbA1c. Data were analyzed through multivariable logistic regression and EBICglasso network analyses. Results: Overall, the prevalence of cardiovascular (CV) biomarker unawareness was 65.5% across participants. Fully adjusted regression models showed that a continuous DDRK score was a significant factor independently associated with lower odds of CV biomarker unawareness (aOR = 0.878, 95% CI [0.824–0.936], *p* < 0.001), followed by a higher MEDAS score (aOR = 0.842, *p* = 0.003) and aging (aOR = 0.961, *p* < 0.001). The network model was dense (sparsity = 0.133) and showed DDRK–CVH literacy as the strongest conditional edge (r_partial_ = 0.202). The conditional associations of BMI (r = 0.000) and physical activity (r = 0.036) with CVH literacy collapsed after conditioning on DDRK and MEDAS. Centrality indicators showed that DDRK had the highest closeness and strength (both = 1.000). Conclusions: DDRK shows strong conditional network associations with Mediterranean diet adherence and CVH literacy. These exploratory findings generate hypotheses for future longitudinal or interventional research.

## 1. Introduction

Nutrition knowledge (NK) refers to the comprehensive concept and process related to the science of nutrition such as dietetics, food, nutrients, food safety and its health outcomes, encompassing diet-disease-related knowledge (DDRK). The study of the association between nutrition knowledge and lifestyle habits has recently become an intensive domain of research, especially in the context of the global epidemic of overweight and obesity [1]. Higher DDRK is believed to sensitize individuals to their health outcomes and to be associated with better lifestyle choices, including smoking cessation, physical exercise, and dietary habits [2,3,4]. Nutrition awareness was examined in the context of various non-communicable disease conditions [5,6], obesity [1,2,7], and dyslipidemia [8] to provide a comprehensive overview of behavioral factors such as physical activity, dietary habits, and smoking in the management of these conditions [9,10,11]. However, while broad cognitive awareness is heavily explored, there is a severe lack of evidence on how DDRK relates to personal cardiovascular biomarker awareness, and more broadly, health literacy.

Better nutrition knowledge has been shown to stem from several sociodemographic determinants, such as education, income, and home residential areas across various regions and demographic contexts. Lower educational levels have been shown to be associated with an inadequate nutrition knowledge score and poor dietary choices in adult populations from four countries of the Arab region [12]. Nutrition knowledge was associated with family income per capita and living area in China [13], while the COVID-19 pandemic highlighted the financial inequality related to NK in Saudi Arabia [14]. Together, these findings suggest that nutrition knowledge varies across populations due to sociodemographic and economic factors, indicating that nutritional intervention should be customized based on the unique regional context.

Nutrition knowledge is consistently associated with better adherence to healthy dietary patterns. Among these, the Mediterranean diet (MD) is the most extensively explored dietary pattern worldwide [15]. Characterized by a high intake of healthy fats, plant-based food, and fatty fish, the MD is widely associated with preventing and managing non-communicable diseases [16]. Across various sociodemographic, economic, and clinical contexts, adherence to the MD is associated with nutrition knowledge levels, as recently observed in a qualitative study in Lebanon [17]. This relationship, mediated through dietary education, has been demonstrated across diverse age groups, including adults [18,19], older adults [20,21], and adolescents [22,23,24].

However, the living conditions specific to each country, regarding individual, social, societal, demographic, and environmental influences, media perceptions and influences, lifestyle behaviors, and clinical and biological determinants, complicate the generalization of the “one-size-fits-all” of these results, which remain context-specific.

During the last decade, Lebanon has witnessed, like many countries globally, a dramatic weakening in compliance with healthy lifestyle habits, with a transition towards poor dietary choices mostly from trans-fat- and added-sugar-dense food, known as the Western diet. This nutrition shift contributes to the high occurrence of non-communicable diseases, including cardiovascular diseases, in the country [25]. Nutrition knowledge has been studied in these various contexts in Lebanon, a country of the Arab region, which is experiencing a continuous shift towards Westernized dietary choices. Major findings in recent years have highlighted multifaceted and complex associations influenced by several determinants, such as the social, demographic, clinical, or lifestyle factors discussed above within specific study groups. Our group previously showed that students compliant with a high adherence to the Mediterranean diet and having better nutrition knowledge awareness had a better academic outcome, as evidenced by a better GPA [26]. Another group found that the nutrition knowledge among patients with type 2 diabetes was associated with better management of the disease through better dietary habits [27]. A nationwide nutrient-specific nutrition knowledge study, focusing solely on the association between trans unsaturated fatty acids and societal behaviors, showed that low awareness and nutrition knowledge concurred with at-risk dietary practices, especially among males [28]. These data observed among adults underscore the practical implications of good nutritional knowledge within this age group. Indeed, evidence from a previous work indicated an intergenerational link between adolescents and their parents, which appears to influence dietary choices and habits, as well as the body weight status of the children [29]. Nutrition knowledge has been shown to be associated with improvements in athletic performance among recreational marathon runners following a five-month dietary educational program [30]. Another aspect investigated regarding nutrition knowledge in Lebanon was the relationship between quality of life and food safety knowledge. In a geographical context of a high risk of foodborne disease, it was found that individuals over the age of 42 with higher education possessed adequate food safety knowledge, which was associated with better practices [31].

Growing global evidence links nutrition knowledge to good dietary habits, health behaviors, and chronic disease outcomes. However, no prior study has applied the DDRK components of the revised GNKQ to simultaneously explore associations with Mediterranean diet adherence, lifestyle factors, and cardiovascular health indicators within the Lebanese context. The present study aimed to examine the structural relationships between lifestyle factors, DDRK, and cardiovascular health metrics among Lebanese adults. Specifically, we sought to: (a) identify the independent lifestyle and sociodemographic predictors of DDRK; (b) determine the behavioral predictors of cardiovascular health literacy and self-reported cardiovascular biomarker unawareness; and (c) characterize the system-level connectedness of these domains using network psychometric analysis.

## 2. Materials and Methods

### 2.1. Ethical Consideration

The present study was conducted in accordance with the ethical principles of the Declaration of Helsinki. Ethical approval was obtained from the Research Ethics Committee of the Higher Centre for Research at Holy Spirit University of Kaslik (approval number: HCR20230503). Prior to accessing the survey, all potential participants were provided with information regarding the research purpose, voluntary participation, and data confidentiality. All participants provided informed electronic consent prior to data collection, and no personally identifiable information was gathered.

### 2.2. Sample and Study Design

**Figure 1:**
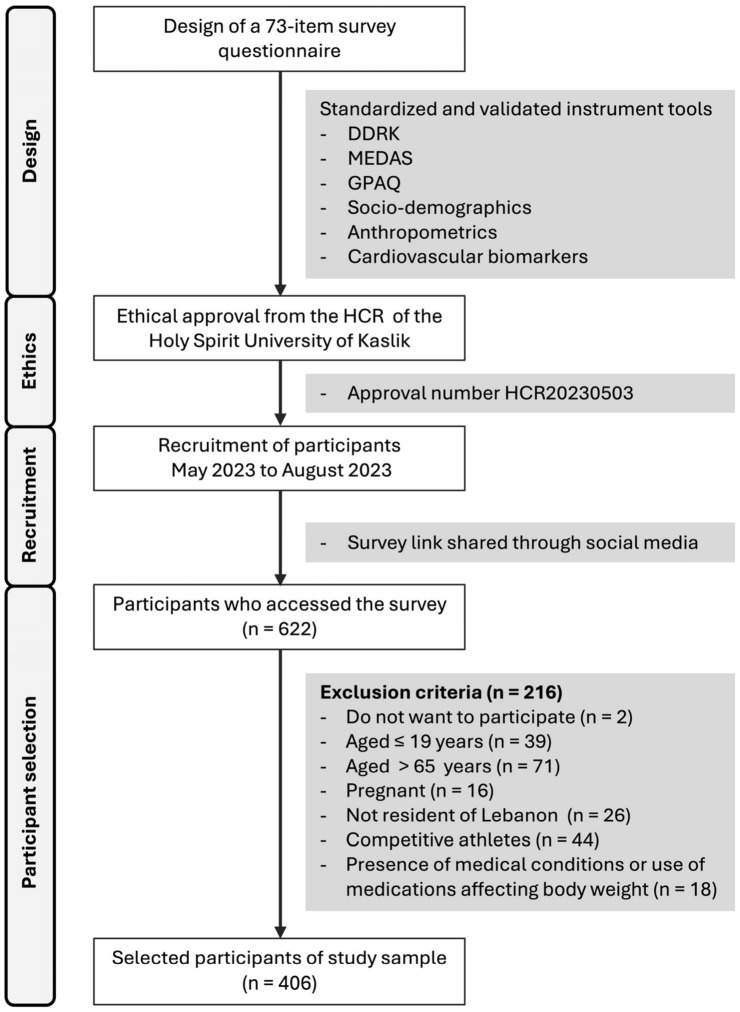
CONSORT-style flow diagram presenting the participant selection process. DDRK, diet-disease-related knowledge (Section 4 of the General Nutrition Knowledge Questionnaire); MEDAS, Mediterranean Diet Adherence Screener; GPAQ, Global Physical Activity Questionnaire.

A cross-sectional, non-probability convenience-sample online survey was conducted. The survey was administered in English. A pilot study was conducted with 22 participants to verify technical functionality and section transitions. After identifying and resolving flaws, these 22 responses were subsequently excluded from the final analysis. A total of 622 participants accessed the electronic survey, of whom 216 were excluded based on the predefined exclusion criteria (Figure 1). Ultimately, 406 participants were included in this study based on the following specific inclusion criteria: provision of informed consent, aged between 20 and 65 years, non-pregnant status, non-athlete status, residency in Lebanon, absence of an active medical condition, non-use of medications affecting body weight (e.g., corticosteroids, weight loss drugs, diuretics, and anti-diuretics), and no current engagement in weight management therapy. This study is reported in accordance with the Strengthening the Reporting of Observational Studies in Epidemiology (STROBE) guidelines for cross-sectional studies (Supplementary File S1) [32].

### 2.3. Study Instruments, Scoring and Data Collection

A self-reported online survey comprising 73 questions on socio-demographic profiles, anthropometric characteristics, lifestyle habits, health-related nutrition knowledge, physical activity, and self-reported lipid and glycemic blood markers was created using Google Forms. Original English validated questionnaires were used as described below. The survey was disseminated through social media platforms in Lebanon as previously described by our group [26,33,34]. Data were collected from May 2023 to August 2023.

#### 2.3.1. Sociodemographic Characteristics and Lifestyle Habits

Participants were instructed to select their age, marital status, living area, education level, employment status, and monthly income. Monthly income was assessed only among employed participants based on skip-logic design: participants reporting no current employment were routed past the income item and classified as “Not employed/No income” to prevent missing income data. Therefore, all 406 participants were assigned to one of the four mutually exclusive income categories. Smoking habits were also assessed.

#### 2.3.2. Body Weight Status

Body mass index (BMI) was estimated from self-reported body weight and height and calculated as weight (kg) divided by height square (m^2^). BMI (in kg/m^2^) was classified into four categories: less than 18.5; between 18.5 and 24.9; more than or equal to 25; and more than or equal to 30 as “underweight”, “normal weight”, “overweight”, and “obese”, based on the World Health Organization criteria and as previously published [34]. To prevent false positive and misclassification as overweight, competitive athletes were excluded.

#### 2.3.3. Mediterranean Diet Adherence

Adherence to the Mediterranean diet (AMD) was assessed using the Mediterranean Diet Adherence Screener (MEDAS), which was originally validated in the Spanish PREDIMED cohort [35] and was previously utilized as a standard proxy in Lebanon [33,36,37,38]. It includes 2 questions on food consumption habits and 12 questions on the frequency of intake of key food items. Each item’s answer has the option “Yes” or “No”, with the response “Yes” scoring 1 point. The maximum score is 14, and the categories of low, fair, and high AMD correspond to the ranges 0 to 5, 6 to 9, and 10 to 14, respectively, in accordance with the original MEDAS scoring framework [39].

#### 2.3.4. Physical Activity Assessment

Physical activity (PA) was assessed and computed using the Global Physical Activity Questionnaires designed by the World Health Organization [40], and widely used to assess PA in the Middle East [41]. Briefly, it consists of 16 questions collecting self-reported information about three domains of PA in daily life, including work, transport, and recreational activities. Each domain is assigned a specific metabolic equivalent (MET) value: 8 METs for vigorous-intensity activity and 4 METs for moderate activity at work and transport. The daily duration of each activity, in minutes, is multiplied by its corresponding MET value. Total PA was calculated in MET-minutes per week into three levels based on the WHO criteria: “highly met” if it exceeds 3000, “moderate” if it exceeds 1500, and “not met” if it falls below 600 MET-minutes per week [42]. In the multivariable regression models, the total MET-minutes per week (PA) was entered as a continuous variable to maximize statistical efficiency. The categorical classification (PA level) was retained for descriptive and bivariate analyses.

#### 2.3.5. Diet-Disease-Related Knowledge (DDRK) Assessment

We utilized the standardized and revised General Nutritional Knowledge Questionnaire (GNKQ), validated for the Arab population [43]. It consists of four independent sections which can be administered individually, covering dietary recommendations, sources of nutrients, healthy food choices, and the diet-disease-related relationship [44]. The independent Section 4, the DDRK subscale, was used in the present study. The DDRK subscale consists of 16 questions: 15 questions are each granted 1 point each for a correct answer, and one complex question comprises six separate items (each awarded 1 point), yielding a total continuous score ranging from 0 to 21, where 21 reflects the highest level of diet-disease-related knowledge (DDRK). Reliability and internal consistency were assessed using Cronbach’s alpha, with values ≥ 0.7 considered acceptable.

#### 2.3.6. Measurement of Self-Reported Cardiovascular Biomarker Unawareness and Cardiovascular Health Literacy

Participants were presented with response options with predefined clinical range categories for each of the five cardiovascular biomarkers and asked to select the range that best corresponded to their most recent laboratory results within the preceding 3 months. The predefined clinical ranges were based on the National Cholesterol Education Program (NCEP) ATP III guidelines for lipids [45] and the American Diabetes Association (ADA) guidelines for HbA1c [46], as follows: total cholesterol < 200 mg/dL (normal), 200–239 mg/dL (borderline high), and ≥240 mg/dL (high); triglycerides < 150 mg/dL (normal), 150–199 mg/dL (borderline high), and ≥200 mg/dL (high); high-density lipoprotein (HDL) > 50 mg/dL (normal) and ≤50 mg/dL (at risk); low-density lipoprotein (LDL) < 100 mg/dL (normal), 100–129 mg/dL (borderline high), and ≥130 mg/dL (high); and glycated hemoglobin (HbA1c): <5.7% (normal), 5.7–6.4% (increased risk/prediabetes), and ≥6.5% (high risk/diabetes ). An “I don’t know” response option was available for all markers.

Cardiovascular biomarker unawareness was evaluated using a binary composite variable derived from these self-reported indicators. Participants were coded as 1 (unaware) if they responded “I don’t know” to one or more of the five primary cardiovascular biomarkers (total cholesterol, LDL cholesterol, HDL, triglycerides and HbA1c) and coded as 0 (aware) if they reported ranged values for all five. The composite variable served as the dependent variable in the binary logistic regression model.

Cardiovascular health literacy (CVH literacy) was assessed as a continuous variable. For descriptive and network analyses, the continuous score was computed by summing the five individual cardiovascular biomarkers total cholesterol, LDL, HDL, triglycerides, and HbA1c, for which participants reported a known clinical range value. This yielded an integer score ranging from 0 (no biomarker range values known) to 5 (all biomarker range values known). Scores were computed in Microsoft Excel using the formula as described in Table S1, where any response other than “I don’t know” was credited as a known value.

### 2.4. Statistical Analysis

Continuous variables (age, MEDAS score, DDRK score, BMI, and MET-minutes per week) and categorical variables (education, income, employment status, sex, region, marital status, and smoking status) were assessed as covariates. Categorical variables were described as frequencies and percentages, and continuous variables as the mean with standard deviation (SD). Data normality was checked by visual observation of Q–Q plots and using Shapiro–Wilk tests. Nonparametric tests were used for continuous variables not meeting a normal distribution. All analyses were conducted in JASP Team (2026). JASP (Version 0.97.1) [Intel]. *p*-values < 0.05 were considered statistically significant.

#### 2.4.1. Bivariate Analyses

Group differences in continuous DDRK scores across all categorical predictor variables were examined using Welch’s *t*-test for two-group comparison (sex) and Welch’s one-way ANOVA (F-test) for multigroup comparisons for all other variables; both tests do not assume equality of variances. Levene’s test was used to confirm equality of variances across subgroups of categorical predictor variables. Effect sizes were reported using Cohen’s d for *t*-tests and eta-squared (η^2^) for F-tests. Spearman correlations (r_s_) were run to quantify associations between continuous variables and DDRK scores, given a non-normal distribution for outcomes.

#### 2.4.2. Regression Analyses

Multivariable linear regression was performed using the DDRK score as the primary dependent variable. Based on bivariate analyses, predictor variables were selected using a lenient screening threshold of *p* < 0.2 to ensure capture of potential confounders. Multicollinearity was evaluated using the variance inflation factor (VIF) with a retention threshold set at VIF < 2.

A multivariable binary logistic regression model was constructed using the nominal composite variable “cardiovascular biomarker unawareness” (0 = aware, 1 = unaware) as the dependent outcome computed from the “I don’t know” self-reported responses for cardiovascular blood markers. A parallel multivariable linear regression was performed using the continuous CVH literacy score (0–5) as a sensitivity analysis to confirm the robustness of the binary logistic regression model.

#### 2.4.3. Network Analysis

A Gaussian Graphical Model (GGM) network analysis was run as the primary integrative analysis utilizing six nodes: the DDRK score (DDRK), MEDAS score (MEDAS), MET-min/week physical activity (PA), BMI, age and cardiovascular health literacy score (CVH literacy). To account for the non-normal and bimodal distribution of the CVH literacy variable, a nonparanormal transformation was applied to the data set prior to network analysis to ensure that the GGM model estimates remained accurate and stable. Network estimation used the extended Bayesian information criterion graphical LASSO algorithm (EBICglasso, γ = 0.5). Nonparametric bootstrapping (2000 iterations) was performed to assess centrality and edge stability. Four centrality indices, betweenness, closeness, node strength, and expected influence, were computed and standardized to z-scores for comparability. The sample size of *n* = 406 was considered adequate for the six-node network analysis, according to guideline recommendations of a minimum of 3 to 5 observations per variable for EBICglasso estimation [47].

#### 2.4.4. Sample Size and Power Justification

A post-hoc sensitivity power analysis was performed to confirm the adequacy of the study sample relative to the primary categorical outcome, cardiovascular biomarker unawareness, based on the bimodal distribution of participants as unaware (*n* = 266) and aware (*n* = 140). An independent-samples *t*-test framework was selected as a conservative proxy to test the continuous DDRK score across the primary outcome based on a two-sided alternative hypothesis with a Type I error rate set at α = 0.05, and a minimal desired power of (1 − β) = 0.90. Sensitivity of the effect size magnitude reliability was considered small-to-moderate as the Cohen’s coefficients fall within the range (0.293 < |δ| ≤ 0.377). A measured δ = 0.339 confirmed that the sample size of *n* = 406 falls securely into the 80–95% power interval to reliably detect effects within this targeted range, supporting the internal validity of the regression and network psychometric analysis.

## 3. Results

### 3.1. Characteristics of the Participants

Table 1 presents the sociodemographic, anthropometric, and lifestyle characteristics of the 406 participants. The mean age was 32.2 ± 10.4 years. Most participants were female (68%), were single (56.7%), and resided in the Mount Lebanon region (62.8%). Regarding education and employment, most participants held a graduate degree (64.8%), and a majority were employed full-time (64.8%) with a monthly salary within the category of middle income for 31.5% of them. Of note, 21.4% of the participants reported no income.

Regarding lifestyle and health variables, 54.2% of the participants were non-smokers. The mean body mass index (BMI) was 25.2 ± 4.2 kg/m^2^ (median: 24.85 kg/m^2^), putting the average score in the overweight range. Specifically, 51.0% fell into the healthy range, while 35% were classified as overweight and 13% as obese.

Physical activity levels were low for 59.1% of the participants, with a mean total MET-minutes/week of 622 ± 797. Additionally, 30.3% reported moderate physical activity and 10.6% reported high activity levels. Adherence to the Mediterranean diet (AMD) was primarily fair (63.0%), with only 14% showing high AMD, and 22.9% showing low AMD. The mean overall MEDAS score was 7.2 ± 2.1 out of 14, while the mean score for diet-disease-related knowledge was 12.5 ± 4.0 out of 21.

### 3.2. Psychometric Reliability of the Diet-Disease-Related Knowledge Questionnaire

As shown in Table 2, reliability analysis across the 16 items of the GNKQ Section 4 yielded a Cronbach’s alpha of 0.76, suggesting an acceptable level of internal consistency. Corrected item-total correlations ranged from 0.25 to 0.47, with individual item omission tracking consistently between 0.73 and 0.77. These diagnostics confirm the structural stability and psychometric validity of the continuous DDRK subscale measurement scale prior to its entry into correlation and multivariable regression models.

### 3.3. Association of Diet-Disease-Related Knowledge Across Sociodemographics and Health Characteristics of Participants

Table 3 shows that significant differences in DDRK were observed based on AMD level (F = 34.169, η^2^ = 0.145, *p* < 0.001), BMI category (F = 25.260, η^2^ = 0.112, *p* < 0.001), PA level (F = 20.371, η^2^ = 0.092, *p* < 0.001) and smoking status (F = 14.750, η^2^ = 0.068, *p* < 0.001) (Table 3). The data indicates higher mean DDRK scores among the never smoker, lower BMI, higher PA and higher AMD groups. Conversely, no significant differences were observed across the subgroups of education levels (*p* = 0.188), sex, marital status, region, employment and income (all *p* > 0.200).

Similarly, as shown in Table 4, Spearman correlation analyses indicated that the continuous DDRK scores were positively associated with MEDAS scores (rs = 0.291, *p* < 0.001) and physical activity as reflected by the total MET-min/week (rs = 0.251, *p* < 0.001). An inverse correlation was found with BMI (rs = −0.245, *p* < 0.001), while the association with age was weak and marginally significant (rs = −0.098, *p* = 0.048). Overall, these correlation coefficients represent modest but statistically significant linear associations among the continuous variables.

### 3.4. Multivariable Linear Regression Factors Associated with Diet-Disease-Related Knowledge

A multivariable linear regression analysis was performed using the DDRK score as the dependent variable. As shown in Table 5, the fully adjusted model demonstrated high statistical significance (F(7, 398) = 9.279, *p* < 0.001), accounting for a modest 12.5% of the total variance in diet-disease-related nutrition knowledge scores (adjusted R^2^ = 0.125, R^2^ = 0.140). Among the examined predictor variables, four distinct lifestyle behaviors emerged as independent, statistically significant determinants of DDRK. The MEDAS score emerged as the strongest independent positive predictor (B = 0.360, β = 0.192, 95% CI [0.183, 0.538], *p* < 0.001). Smoking status was the second most important predictor. Never-smoking compared to current-smoking individuals scored significantly higher on the continuous DDRK scale (B = 1.487, *p* < 0.001). Physical activity levels also demonstrated a significant independent positive association (B = 0.0006, *p* = 0.016). Conversely, BMI retained a significant independent inverse relationship (B = −0.094, *p* = 0.046), indicating that higher body weight profiles track with lower knowledge baselines.

### 3.5. Cardiovascular Health Literacy and Self-Reported Awareness

**Figure 2:**
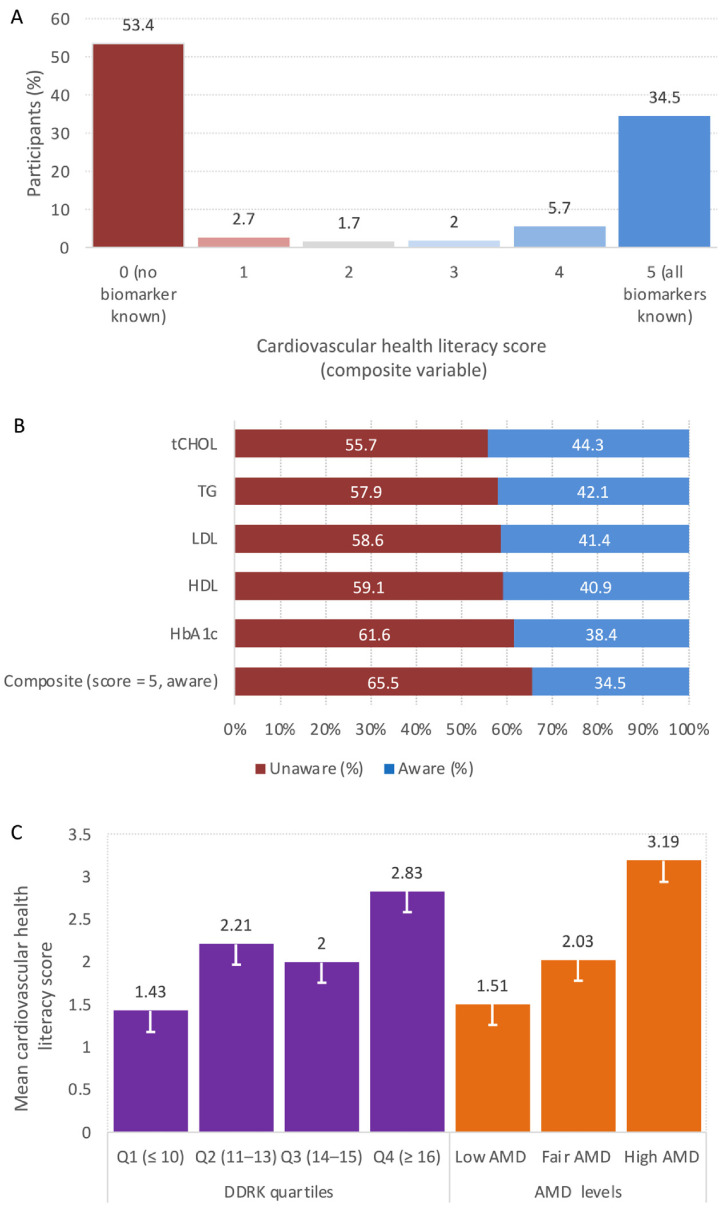
Cardiovascular self-reported biomarker awareness and its associations with cardiovascular literacy and adherence to the Mediterranean diet among participants (*n* = 406). (**A**), The stacked bar illustrates the percentage of participants unaware (red) versus those aware (blue) of their individual cardiovascular biomarker values, including glycated hemoglobin (HbA1c), triglycerides (TGs), high-density lipoprotein (HDL), low-density lipoprotein (LDL), and total cholesterol (tCHOL). The composite score captures participants who have at least one biomarker unknown (score ≤ 4, unaware). Conversely, the aware composite group score five exactly. (**B**), Distribution of the cardiovascular health literacy score (0–5), calculated as the total number of biomarkers known as described in (**A**). (**C**) Mean cardiovascular health literacy across DDRK quartiles and AMD levels. DDRK, diet-disease-related knowledge; AMD, adherence to the Mediterranean diet.

Figure 2 and Table S2 present the cardiovascular health literacy (CVH literacy) profile of the study sample. As shown in Figure 2A, individual cardiovascular biomarker unawareness ranged from 55.7% (total cholesterol) to 61.6% (HbA1c), with HDL, triglycerides, and LDL falling in this range. Overall, 65.5% of participants were classified as lacking awareness of at least one of these five cardiovascular biomarkers (composite variable, score ≤ 4), while only 34.5% reported knowing all five biomarker values (score = 5). The distribution of CVH literacy is strongly bimodal (Shapiro–Wilk *p* < 0.001), as illustrated in Figure 2B, where 53.4% of participants scored zero and 34.5% scored five, with only 12.1% scoring between one and four. This indicates that most participants either knew all or none of their CV biomarkers’ values. Both the DDRK knowledge quartile and Mediterranean diet adherence (AMD) levels were significantly associated with the CVH literacy score (Figure 2C). Mean scores increased progressively across DDRK quartiles, from 1.43 ± 0.19 in Q1 to 2.83 ± 0.24 in Q4 (H = 18.549, *p* < 0.001). Similarly, mean scores increased across AMD levels from 1.51 ± 0.22 (low AMD) to 3.19 ± 0.31 (high AMD) (H = 19.641, *p* < 0.001), indicating that higher diet-disease-related knowledge and higher AMD were both associated with greater CVH literacy.

### 3.6. Multivariable Logistic Regression Predicting Cardiovascular Biomarker Unawareness

A multivariable binary logistic regression model was further performed to determine independent factors associated with CV biomarker unawareness (Table 6). The fully adjusted model demonstrated a highly significant statistical fit (∆χ^2^ = 49.065, *p* < 0.001), accounting for 15.6% of the total variance in CV biomarker unawareness (Nagelkerke (R^2^ = 0.156)). Three parameters emerged as highly significant independent predictors of a lower likelihood of CV biomarker unawareness. The DDRK score maintained a significant inverse association with CV biomarker unawareness (B = −0.130, aOR = 0.878, *p* < 0.001, 95% CI: [0.824, 0.936]), indicating that for each additional point on the continuous DDRK subscale, the odds of remaining unaware of self-reported metabolic risk markers dropped by 12.2%. Similarly, the MEDAS score significantly reduced the likelihood of CV biomarker unawareness, with each one-point increase on the continuous MEDAS score lowering the odds of a lack of awareness by 15.8% (B = −0.172, aOR = 0.842, *p* = 0.003, 95% CI: [0.753, 0.942]). Participant age was also significantly inversely associated with CV biomarker unawareness (B = −0.040, aOR = 0.961, *p* < 0.001), lowering the odds of unawareness by 4.0% per year. Sex (*p* = 0.879), BMI (*p* = 0.651), education level and smoking statuses showed no independent associations with the lack of CV biomarker unawareness. To confirm the robustness and consistency of these findings, a parallel sensitivity analysis was conducted by treating the CVH literacy score as the dependent variable via multivariable linear regression (Table S3).

### 3.7. Network Analysis

Altogether, the preceding analyses showed pairwise associations between DDRK, MEDAS, PA, BMI, age, and cardiovascular health literacy (CVH literacy). To further explore the interdependency and connectedness among these variables simultaneously, a partial correlation network analysis was performed using the EBICglasso estimator. Our analysis revealed a dense network, retaining 13 of 15 possible edges as non-zero (*n* = 406, γ = 0.50, sparsity = 0.133), indicating that 86.7% of all possible pairwise conditional associations were active after regularization (Table S4). To ensure our model was robust against a non-normal distribution based on the bimodal profile of the CVH literacy score, a sensitivity analysis was run, showing stable parameter matrices (Table S5).

**Figure 3:**
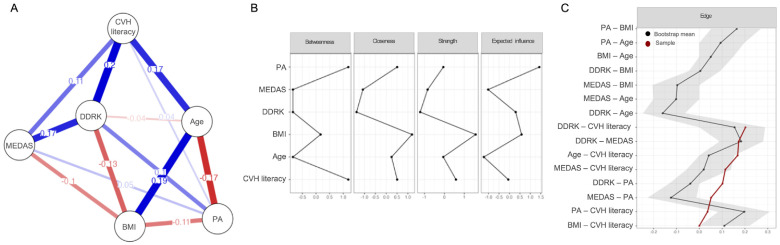
Regularized EBICglasso network psychometric topology and node centrality profiles across continuous dietary, behavioral, and health literacy domains (*n* = 406). (**A**) Estimated Gaussian Graphical Model (GGM) network plot using the EBICglasso estimator. Nodes represent the six continuous variables, and edges represent regularized partial correlation weights (blue lines indicate positive pathways, red lines indicate negative pathways); each edge represents the unique association between two variables after controlling for all other variables in the network, where the regularization process removes weak or spurious associations to prevent false positives. (**B**) Centrality measures plot demonstrating the relative standardized indices for betweenness, closeness, node strength, and expected influence. (**C**) Centrality statistics grid detailing the raw values for node strength, betweenness, and closeness across all 2000 bootstrap simulations, establishing high structural stability. DDRK, diet-disease-related knowledge; MEDAS, Mediterranean Diet Adherence Screener; BMI: body mass index (kg/m^2^); PA, physical activity assessed by MET-min/week (metabolic equivalent minutes per week); CVH literacy, cardiovascular health literacy; Age, in years.

Figure 3A visually maps the conditional interdependencies across the six nodes. The strongest positive partial correlation in the entire network was observed between DDRK and CVH literacy (r_partial_ = 0.202), followed by the age–BMI (r_partial_ = 0.193), DDRK–MEDAS (r_partial_ = 0.172), age–CVH literacy (r_partial_ = 0.168), and DDRK–PA (r_partial_ = 0.102) edges (Table S4). The DDRK–CVH literacy edge remained the strongest after conditioning on BMI, age, PA, and MEDAS, confirming that the association between DDRK and cardiovascular health literacy was direct and independent of all remaining network variables. DDRK also showed a significant negative partial correlation with BMI (r_partial_ = −0.125), while BMI exhibited negative associations with PA (r_partial_ = −0.106) and MEDAS (r_partial_ = −0.096). Notably, the BMI–CVH literacy and PA–CVH literacy edges were fully regularized to zero or near zero, respectively, reflecting that the associations of adiposity and PA with CVH literacy are fully attenuated when the model conditions on DDRK and MEDAS (Figure 3A and Table S6).

Figure 3B and Table S7 confirm that DDRK was the most strongly and directly connected node in the network with the highest closeness (1.000) and strength (1.000) centrality. CVH literacy had the highest expected influence (1.000) and shared the highest betweenness centrality with age (both = 1.000), suggesting that it occupies a central position connecting the other nodes in the network topology. Table S8 further characterized the connectivity patterns, where the MEDAS showed the highest Barrat (1.527) and Zhang (1.340) clustering coefficients, indicating strong localized clustering around this node. Finally, the bootstrapped stability curves in Figure 3C proved that estimated edge weights and centrality profiles are robust against sampling variations, validating our interpretation. Together, these findings support the interpretation that DDRK occupied a central role in the network of associations linking the MEDAS, PA, BMI, and CVH literacy in our study sample.

Taken together, all these results reveal a consistent and convergent pattern that DDRK is systematically associated with healthier lifestyle habits at the bivariate level, remains an independent correlate in multivariable regression models, and shows the highest closeness and strength centrality in the network topology. These complementary analytical perspectives provide the interpretive framework for the discussion below.

## 4. Discussion

This study presents an original, comprehensive network evaluation of diet-disease-related knowledge (DDRK), lifestyle behaviors such as adherence to the Mediterranean diet (AMD), assessed by the Mediterranean Diet Adherence Screener (MEDAS), smoking, physical activity (PA), and the cognitive domain of cardiovascular health (CVH) literacy. By combining conventional multivariable regression with a regularized Gaussian Graphical Model network approach, we identified DDRK as the most topologically central node within the conditional association network linking lifestyle variables and self-reported cardiovascular biomarkers. From this analysis, three main findings emerged. First, DDRK was independently and positively associated with a higher AMD and PA levels, and the non-smoking status, and inversely with the body mass index (BMI). Second, DDRK, MEDAS scores, and aging emerged as the strongest independent predictors of a lower likelihood of self-reported CV biomarker unawareness. Third, DDRK occupied the highest closeness and strength centrality within the network topology, while BMI and PA showed no direct partial association with CVH literacy after accounting for the MEDAS and DDRK.

We built our research based on the health literacy taxonomy introduced by Nutbeam [48], which classifies health-related cognitive capacities into functional (declarative/recall skills) and interactive/critical (information appraisal and behavioral application) tiers. Indeed, our work distinguishes between DDRK and CVH literacy as two distinct, non-hierarchical constructs. In our study, self-reported CV biomarker values are captured by the CVH literacy that is conceptualized specifically through its functional dimension, representing a form of functional literacy applied to personal biometric monitoring, while the capacity to interpret and apply health information to active lifestyle behavior is assessed by the continuous DDRK score (representing critical health literacy). Together, these two independent domains contribute to a multidimensional health literacy profile. Interestingly, our empirical findings indicate that DDRK and biomarker awareness are related but not interchangeable. Each additional point in the DDRK score reduced the odds of biomarker unawareness by 12.2%, while 65.5% of our participants who were predominantly university-educated could not identify their personal CV biomarker values. This discrepancy underscores the fact that nutrition knowledge does not automatically confer personal biomarker awareness or functional health literacy.

The healthcare landscape in Lebanon can explain this awareness disconnect. In this context, patients frequently bypass physicians to receive their blood test results directly. They instead receive them from laboratories, online portals, and digital copies. Consequently, while participants, even those holding a high education level, can recall a “high cholesterol“ status due to the asterisk and bold text labels on their laboratory reports, they frequently lack the clinical information necessary to implement actionable dietary modifications, which may reflect poor CV health literacy. This highlights how, in the Lebanese context, a high educational status does not protect against low health literacy, because individuals navigate unguided laboratory portals without active interpretation and diagnosis provided by healthcare practitioners. Regarding the objectivity of these data compared to clinical measurements, chronic disease validation studies have shown that categorical self-reporting of established laboratory values, including glycemic and lipid markers, correlates strongly with verified clinical metrics [49,50,51]. Our use of predefined ranges and an explicit “I don’t know” option minimized recall artifacts, making this baseline a reliable proxy for objective cardiovascular biomarker unawareness. This disconnect between recognizing biomarkers and lacking health knowledge has been defined as the “knowledge–behavior gap” [52], which appears to be more prevalent among the youth, creating a substantial public health burden for the next decades. Furthermore, because our study used an online convenience sample, our findings may principally capture participants with regular internet access centered in urban areas. Therefore, this paradox may not represent rural or economically disadvantaged areas of Lebanon where healthcare access is even more limited.

This interpretation is strongly reinforced by the bimodal, “all-or-nothing” pattern of CVH literacy observed in our sample. This distribution may reflect the online recruitment method of a population with internet access, which tends to be higher among people with higher education. This could explain the decreased variance associated with CVH literacy in our model. To place these findings in a global context, others have explored the association between nutritional knowledge and sociodemographic characteristics worldwide, such as those in Singapore [53], Morocco [54], Turkey [55], the United Arab Emirates [56], and Poland [57]. Surprisingly, our Lebanese sample presents an “education paradox”. Education is commonly assessed based on the highest level attained, as it is often theorized to shape social identity and foster positive attitudes toward lifestyle habits like smoking cessation, exercise, limited social media, and healthier dietary choices [58,59,60]. Surprisingly, having a high education level did not guarantee better CVH literacy or healthier lifestyle choices. Indeed, a large majority of participants held a university degree, yet many of them exhibited poor lifestyle habits, including high rates of smoking, overweight or obesity, and low physical activity. When interpreting this paradox, it is important to note that our reliance on an online convenience sample likely underrepresents individuals with the lowest digital literacy and least access to healthcare. This is precisely the subgroup where CVH literacy deficits may be most critical for cardiometabolic risk. Furthermore, reporting high cholesterol levels was a strong, independent negative factor linked to a higher DDRK score. Given the self-reported nature of the data, it is possible that while participants were aware of their high cholesterol, they may, however, not understand the dietary modifications required to manage it. Our data suggest that within the highly educated convenience sample, participants’ lifestyle habits were the drivers of health literacy rather than socioeconomic demographics.

When examining factors associated with good DDRK, AMD emerged as the strongest independent positive predictor of DDRK, consistent with previous studies that have observed a positive linear relationship between these two variables across diverse populations and age groups [17,18,19,20,21,22,23,24]. This finding aligns with previous Lebanese reports from our group, where Lebanese University students exhibited a better GPA along with a higher AMD [26], and others, who showed that better dietary knowledge was associated with better food choices in Lebanese adults [17,27]. The observed gradient from low to high AMD across the DDRK quartiles mirrors and confirms this dose–response-like relationship at the population level. This suggests that changes in lifestyle behaviors, encompassing primarily dietary changes, should focus on the core pillars of the traditional Mediterranean diet.

Smoking status was the second most important predictor of DDRK. Smoking status is a long-standing social determinant negatively associated with health behaviors and health status [61,62]. Nicotine dependence has been associated with lower compliance across multiple dimensions of nutrition literacy, as recently reported in an Iranian cohort [63]. In the Lebanese context, smoking is deeply embedded in cultural practices and has a high prevalence among groups with lower education levels. These results reinforce the importance of implementing educational initiatives focused on the risks of smoking, especially regarding cardiovascular disease. Notably, recent evidence has demonstrated that despite the substantial immediate health benefits of smoking cessation, the risk for chronic disease remains in the long-term for about two decades [64]. This finding underscores that smoking cessation programs in Lebanon represent a clinical necessity rather than a complementary approach to healthcare.

Furthermore, physical activity (PA) exhibited a significant positive association with DDRK. Intriguingly, moderate PA exhibited a significant positive association with higher DDRK, while high PA did not. We hypothesize that the lack of association in this latter group may be due to its low frequency within our sample (10.6%), where participants may engage in high PA for occupational reasons rather than health-driven motivation. In contrast, people with moderate leisure-time activity may be more prone to exercise for well-being and longevity reasons and are inherently more engaged with nutrition literacy. Our observation concurs with prior evidence indicating that PA and nutrition literacy are associated, based on overall health motivation [2].

While our logistic regression models looked at independent predictors separately, the regularized network analysis shows the interconnectedness across the same variables simultaneously. Centrality analysis shows that DDRK occupied the most central position in the network, having the highest closeness and node strength. Conceptual CVH literacy is therefore not just abstract knowledge; it potentially positions CVH literacy as an important cognitive factor correlating with personal health vigilance. While directionality cannot be confirmed from the cross-sectional design of our study, our findings suggest that monitoring health biomarkers without teaching patients how dietary habits connect with the risk of diseases may result in this being less well-integrated with patient knowledge, especially among young people. Indeed, we found that age was also independently and inversely associated with CV biomarker unawareness. This reflects clinical realities where older individuals are more likely to know their laboratory values, possibly reflecting routine clinical monitoring associated with aging, regardless of their lifestyle habits, including dietary habits.

The most notable finding from the network topology was the collapse to zero or near-zero of the edges between body mass index (BMI), PA, and CVH literacy. Being overweight, obese or inactive, with a higher risk of chronic diseases, does not reflect a better vigilance of health indicators. These findings are alarming as they demonstrate that body weight status does not dictate a participant CV biomarker awareness. Taking into account the epidemic of comorbidity linked to obesity and sedentary behavior among young people, a patient with obesity is statistically no more likely to know their numbers than a person with a healthy BMI. This observation reinforces our interpretation regarding public health interventions, which should imperatively target the structural support of the network, focus on the cognitive perspective of the patient, and assess their contemplative attitude to prevent ineffective lifestyle interventions.

This connection between knowledge and behavior is happening during a serious Lebanese environmental crisis. However, while good dietary habits may encourage education toward higher DDRK, our findings indicate a lower prevalence of high adherence and a shift toward fair- to-low AMD in the Lebanese population, a trend that we have previously observed [26,33,34], as well as others [65,66,67]. This shift can no longer be solely attributed to the social and economic collapse the country has faced over the last decade [68]. In addition to food insecurity, poor-quality food, and decreased purchasing power, Lebanon, like many countries worldwide and particularly in the Arab region [69], has adopted a modern lifestyle characterized by the “Westernization” of dietary habits, especially among children, adolescents, and young adults [70,71]. In line with previous observations, low AMD has been associated with dyslipidemia, especially high cholesterol levels, and cardiovascular risks in Arab countries [69,72,73]. Paradoxically, while epidemiologic studies on chronic diseases have promoted better dietary habits, other studies have observed a decline in healthy eating habits in favor of energy-dense foods [74,75,76]. These observations illustrate a nutritional paradox in the pursuit of better health outcomes for nutrition-related diseases, complicating the global public health strategies. However, without adequate DDRK, lasting healthy habits are unlikely to occur.

### Limitations and Strengths of the Study

The observational nature of the data presented in this study has several limitations. First, the cross-sectional design does not allow for the determination of causality regarding the etiological factors contributing to cardiovascular risk outcomes. Second, due to an economic crisis that caused the closure of biomedical industries, planned laboratory measurements were cancelled. Consequently, the results were based on self-reported information, which introduced potential recall bias in our study. Recalling the most recent blood lipid and glycemic information from predefined ranges is less accurate than clinical measurements, and using self-reported height and weight to estimate BMI may differ from the BMI calculated through direct anthropometric measurements. Third, convenience sampling obtained via digital social media platforms introduced selection bias, likely restricting the sample to those with internet access and overrepresenting single participants, or underrepresenting several regions more severely impacted by the economic crisis, such as South Lebanon, Bekaa, and North Lebanon. Therefore, our data, although representing a diverse sample of the target population, may be not entirely generalizable to the whole country; nevertheless, the identified associations and predictors obtained from the standardized instruments provide a robust basis for further investigation. Although the MEDAS has not been formally validated in Lebanon, its selection was justified for the consistency of results based on our previous studies [33,38] and its widespread recognition across various Arab countries [36,77,78,79,80]. Cardiovascular biomarker awareness was assessed by asking participants to select their most recent laboratory result from predefined clinical range categories, with an explicit “I don’t know” option provided to minimize social desirability bias. While this design is more robust than simple yes/no awareness questions, residual misclassification of clinical categories cannot be excluded, as participants may have imprecisely recalled their laboratory results. Importantly, such misclassification would not affect the primary CVD literacy score, which credits any non-”I don’t know” response equally regardless of the specific category selected. Verification of self-reported biomarker values against laboratory records was not feasible in this community-based survey design and represents a direction for future research. Self-reported biomarker awareness may be subject to social desirability bias, whereby participants selected a clinical category despite genuine uncertainty. This would result in conservative underestimation of true biomarker unawareness prevalence; the reported 65.5% composite ignorance rate should therefore be interpreted as a lower-bound estimate of cardiovascular health literacy deficits in this sample.

Notwithstanding these limitations, our study presents notable strengths. It is the first application on the adult Lebanese population that has integrated three validated instruments (DDRK, MEDAS, and GPAQ) with self-reported cardiovascular health indicators. A major methodological strength of this study is the high stability and adequacy of our sample size. Despite utilizing a convenience sampling approach, post-hoc metrics confirmed that our sample size of *n* = 406 provided excellent analytical power. Specifically, the events-per-variable (EPV) ratio reached 29.6 for the multivariable logistic regression, exceeding the stringent recommended threshold of EPV ≥ 10 to eliminate coefficient bias [81,82]. Furthermore, our sample size offered a highly sufficient observations-to-node ratio for the network analysis, ensuring robust psychometric stability, as empirically demonstrated by our bootstrap stability parameters. The identification of small-to moderate effect sizes and the statistically significant association between social determinants and lifestyle factors provide a good-to-moderate model fit. This framework delivers a valuable reference baseline profile for DDRK in Lebanon, and our final models offer a reproducible structure for future longitudinal studies. The inclusion of an explicit “I don’t know” response option for each biomarker item, rather than forcing selection among clinical categories, represents a methodological strength that reduces social desirability bias and provides a valid behavioral indicator of cardiovascular health literacy as a self-reported construct.

## 5. Conclusions

This study demonstrates that diet-disease-related knowledge (DDRK) is systematically associated with healthier lifestyle behaviors among Lebanese adults, including adherence to the Mediterranean diet, higher physical activity levels, and non-smoking status. Individuals with higher DDRK also display greater awareness of their cardiovascular health indicators, as reflected by lower rates of “I don’t know” responses to self-reported cardiovascular biomarker values. By moving beyond standard linear models to a regularized network psychometrics framework, our findings reveal that DDRK occupies the most interconnected position in the network, holding the highest node strength and closeness centralities. Lebanon, like other countries of the Arab region, is experiencing the effects of dietary Westernization, a transition that is associated with a regional nutritional paradox: the rising awareness of chronic disease risk coexisting with a decline in healthy dietary habits. Importantly, the finding that physical factors such as body mass index and physical activity show zero or near-zero conditional associations with cardiovascular health (CVH) literacy suggests that behavioral changes may operate through a cognitive framework. Addressing this paradox highlights CVH literacy and DDRK as important elements to consider within any public health strategy aimed at reducing the burden of non-communicable diseases, especially cardiovascular diseases. Future interventional studies should consider physical activity, smoking cessation, and nutrition literacy centered on the Mediterranean diet as potential targets, evaluating whether leveraging their central positioning within the exploratory network of associations can help improve CVH literacy over the long term. Altogether, these findings warrant confirmation in longitudinal and interventional study designs.

## Figures and Tables

**Table 1:** Baseline sociodemographic characteristics of the study sample (*n* = 406).

Categorical Variables	Frequency	Percent
Sex		
Female	276	68.0
Male	130	32.0
District		
Beirut	55	13.5
Beqaa	35	8.6
Mount Lebanon	255	62.8
North Lebanon	36	8.9
South Lebanon	25	6.2
Marital status		
Single	230	56.7
Married	165	40.6
Divorced/Separated/Widowed	11	2.7
Employment		
Full-time	263	64.8
Part-time	56	13.8
Not employed/retired	87	21.4
Education level		
Undergraduate and below	65	16.0
Graduate	263	64.8
Postgraduate	78	19.2
Monthly income		
Not employed/No income (skip-logic tier)	87	21.4
Low income (<$500/month)	95	23.4
Middle income ($500–$1000/month)	128	31.5
High income (>$1000/month)	96	23.6
Smoking status		
Current smoker	158	38.9
Never smoker	220	54.2
Ex-smoker	28	6.9
Perceived BMI (kg/m^2^)		
Underweight	4	1.0
Healthy	207	51.0
Overweight	142	35.0
Obese	53	13.0
AMD levels ^1^		
Low	93	22.9
Fair	256	63.0
High	57	14.0
PA level (MET-min/week)		
Low	240	59.1
Moderate	123	30.3
High	43	10.6
**Continuous Variables**	**Mean**	**SD**
Age	32.2	10.4
Perceived BMI ^2^	25.2	4.2
MEDAS score ^3^	7.2	2.1
DDRK score ^4^	12.5	4.0
MET-min/week (PA) ^5^	622	797

^1^ AMD: Adherence to the Mediterranean diet; ^2^ BMI: body mass index computed based on self-reported weight and height metrics; ^3^ MEDAS: Mediterranean Diet Adherence Screener; ^4^ DDRK: diet-disease-related knowledge score; ^5^ MET-min/week: metabolic equivalent minutes per week; PA: physical activity; SD: standard deviation.

**Table 2:** Internal consistency and psychometric reliability analysis of the diet-disease-related knowledge questionnaire.

Key Nutrition and Health Concepts (16 Items)	Corrected Item-Total Correlation	Cronbach’s Alpha If Item Deleted
Diseases related to low fiber	0.47	0.73
Diseases related to sugar intake	0.35	0.74
Diseases related to salt (or sodium) intake	0.44	0.73
Recommendations to prevent cancer	0.29	0.75
Recommendations to prevent heart disease	0.40	0.74
Recommendations to prevent diabetes	0.46	0.73
Foods that raise blood cholesterol	0.36	0.74
High glycemic index foods	0.46	0.73
Cutting fat for weight loss	0.25	0.75
High protein diet for weight loss	0.25	0.75
Bread consumption and weight gain	0.43	0.74
Fiber and body weight management	0.26	0.75
Behavioral strategies to maintain body weight	0.36	0.77
Body mass index of 23 kg/m^2^	0.42	0.74
Body mass index of 31 kg/m^2^	0.36	0.74
Body shape and cardiovascular risk	0.33	0.74
**Reliability Measure**	**Items Number**	**Value**
Overall internal consistency (Cronbach’s Alpha)	16	0.76

**Table 3:** Bivariate comparisons of the diet-disease-related knowledge scores across the categorical variables characterizing the participants (*n* = 406).

Variable/Subgroup	*n*	Mean DDRK ^1^ ± SD	Test Statistic ^#^	*p*-Value	Effect Size
Sex			t = 0.901	0.368	d = 0.091
Female	276	12.64 ± 4.31			
Male	130	12.28 ± 3.35			
Marital status			F = 1.740	0.419	η^2^ = 0.009
Single	230	12.50 ± 4.40			
Married	165	12.66 ± 3.48			
Divorced/Widowed/Separated	11	11.09 ± 3.33			
District/Region			F = 3.343	0.502	η^2^ = 0.008
Beirut	55	12.13 ± 4.15			
Beqaa	35	13.06 ± 3.92			
Mount Lebanon	255	12.41 ± 4.01			
North Lebanon	36	12.86 ± 3.75			
South Lebanon	25	13.36 ± 3.88			
Education level			F = 3.344	0.188	η^2^ = 0.012
Undergraduate	65	12.48 ± 3.95			
Graduate	263	12.35 ± 4.12			
Postgraduate	78	13.15 ± 3.65			
Employment status			F = 1.733	0.420	η^2^ = 0.009
Full-time employment	263	12.28 ± 4.10			
Part-time employment	56	13.00 ± 3.85			
Not employed	87	12.95 ± 3.92			
Income category			F = 1.920	0.589	η^2^ = 0.007
Not employed/No income	87	12.95 ± 3.92			
Low income	95	11.91 ± 4.15			
Middle income	128	12.58 ± 3.88			
High income	96	12.68 ± 3.72			
Smoking status			F = 14.750	<0.001	η^2^ = 0.068
Never smoker	220	13.45 ± 3.82			
Current smoker	158	11.24 ± 4.12			
Ex-smoker	28	12.54 ± 3.75			
BMI category ^2^			F = 25.260	<0.001	η^2^ = 0.112
Underweight	4	13.50 ± 2.85			
Healthy weight	207	13.35 ± 3.62			
Overweight	142	11.83 ± 4.01			
Obese	53	11.08 ± 4.15			
PA level ^3^			F = 20.371	<0.001	η^2^ = 0.092
Low	240	11.86 ± 4.21			
Moderate	123	13.57 ± 3.65			
High	43	13.23 ± 3.88			
AMD ^4^			F = 34.169	<0.001	η^2^ = 0.145
Low	93	10.60 ± 4.35			
Fair	256	12.90 ± 3.82			
High	57	13.98 ± 3.42			

^1^ DDRK, diet-disease-related knowledge score; ^2^ BMI, body mass index; ^3^ PA, physical activity; ^4^ AMD, adherence to the Mediterranean diet. ^#^ Welch’s *t*-test statistic reported for two-group comparisons (sex), and Welch’s F-test reported for multi-group comparisons. Effect sizes are reported as Cohen’s d for *t*-tests and eta-squared (η^2^) for F-tests.

**Table 4:** Spearman correlation matrix among continuous variables (*n* = 406).

Variable	DDRK Score ^1^	MEDAS Score ^2^	MET-min/Week (PA) ^3^	BMI ^4^	Age
DDRK Score ^1^	—				
MEDAS Score ^2^	0.291 ***	—			
PA (MET-min/week) ^3^	0.251 ***	0.223 ***	—		
BMI (kg/m^2^) ^4^	−0.245 ***	−0.197 ***	−0.241 ***	—	
Age	−0.098 *	−0.038	−0.251 ***	0.311 ***	—

Values are Spearman correlation coefficients (r_s_). *** *p* < 0.001; * *p* < 0.05. Diagonal entries are self-correlations (—). ^1^ DDRK, diet-disease-related knowledge; ^2^ MEDAS, Mediterranean Diet Adherence Screener; ^3^ MET-min/week, metabolic equivalent minutes per week; PA, physical activity; ^4^ BMI, body mass index.

**Table 5:** Multiple linear regression predicting diet-disease-related knowledge score (*n* = 406).

Predictor Variable	B	SE	β	t-Value	95% CI	*p*-Value	VIF
Model Summary: R^2^ = 0.140|Adjusted R^2^ = 0.125|F(7, 398) = 9.279, *p* < 0.001							
Intercept	10.998	1.503	—	7.317	[8.043, 13.952]	<0.001	—
MEDAS score	0.360	0.09	0.192	3.992	[0.183, 0.538]	**<0.001**	1.033
BMI (kg/m^2^)	−0.094	0.047	−0.099	−1.998	[−0.187, −0.001]	**0.046**	1.069
PA (MET-min/week)	0.0006	0	0.116	2.411	[0.000, 0.001]	**0.016**	1.033
Smoking status ^a^							
Never smoker	1.487	0.419	—	3.552	[0.664, 2.311]	**<0.001**	1.033
Ex-smoker	0.871	0.777	—	1.12	[−0.657, 2.399]	0.263	1.033
Education level ^b^							
Postgraduate	0.313	0.492	—	0.636	[−0.654, 1.280]	0.525	1.007
Undergraduate	0.048	0.523	—	0.091	[−0.980, 1.075]	0.927	1.007

^a^ Reference category: Current smoker. ^b^ Reference category: Graduate education. B: Unstandardized coefficient; SE: standard error; β: standardized coefficient; VIF: variance inflation factor (all < 2.0, no multicollinearity concern). Significant predictors (*p* < 0.05) are highlighted in bold; MEDAS, Mediterranean Diet Adherence Screener; BMI, body mass index; MET-min/week, metabolic equivalent minutes per week; PA, physical activity.

**Table 6:** Multivariate binary logistic regression predicting cardiovascular biomarker unawareness (*n* = 406).

Predictor Variable	B	SE (B)	aOR	95% CI (aOR)	*p*-Value
Model Summary: Nagelkerke R^2^ = 0.156|−2LL = 480.161|Hosmer–Lemeshow *p* = 0.955| Δχ^2^(9) = 49.065, *p* < 0.001					
Intercept	5.111	1.077	165.789	[20.071, 1369.464]	<0.001
DDRK score	−0.130	0.032	0.878	[0.824, 0.936]	**<0.001**
MEDAS score	−0.172	0.057	0.842	[0.753, 0.942]	**0.003**
Age (years)	−0.040	0.011	0.961	[0.941, 0.982]	**<0.001**
BMI (kg/m^2^)	−0.014	0.03	0.986	[0.929, 1.047]	0.651
Sex (Male) ^a^	−0.038	0.25	0.963	[0.589, 1.572]	0.879
Smoking status ^b^					
Never smoker	0.262	0.256	1.299	[0.787, 2.145]	0.306
Ex-smoker	0.020	0.46	1.021	[0.415, 2.512]	0.965
Education level ^c^					
Postgraduate	−0.472	0.284	0.624	[0.357, 1.088]	0.097
Undergraduate	0.100	0.313	1.105	[0.599, 2.040]	0.749

^a^ Reference category: Female. ^b^ Reference category: Current smoker. ^c^ Reference category: Graduate education. B, unstandardized coefficient; SE, standard error; aOR, adjusted odds ratio; CI, confidence interval; DDRK, diet-disease-related knowledge; MEDAS, Mediterranean Diet Adherence Screener; BMI, body mass index. Significant predictors (*p* < 0.05) are highlighted in bold.

## Data Availability

The original data presented in the study are openly available in the Open Source Framework repository at https://osf.io/57unh/overview?view_only=fc51b78c45eb49e1ad31519d60a8c753 (accessed on 29 June 2026).

## Supplementary Materials

The following supporting information can be downloaded at: https://www.mdpi.com/article/10.3390/nu18132196/s1, Table S1. Operational Definitions and Scoring Formulas for Cardiovascular Health Variables. Table S2. Cardiovascular biomarker awareness and cardiovascular health literacy score profile of the study sample (*n* = 406). Table S3. Multivariate linear Regression predicting cardiovascular health literacy score (*n* = 406). Table S4: Global network architecture and sparsity parameters. Table S5. Sensitivity Analysis Network Parameters: Nonparanormal Regularized Partial Correlation Weights Matrix and Standarized Node Centrality Indicators. Table S6: Regularized partial correlation edge weights matrix across the 6 network nodes. Table S7: Centrality measures per variable. Table S8: Local network clustering coefficients per variable. File S1. STROBE Statement—Checklist of items that should be included in reports of observational studies.
