# Supplementary material for: Lifestyle Factors and Diet-Disease-Related Knowledge: A Network Psychometric Analysis of Cardiovascular Health Literacy Among Lebanese Adults
Source: Nutrients. 2026 Jul 6;18(13):2196. doi: 10.3390/nu18132196 (PMC13364409; doi:10.3390/nu18132196)
Supplement: Supplementary file 1 [file nutrients-18-02196-s001.zip › nutrients-4396486-File S1.pdf]

STROBE Statement — Checklist of items that should be included in reports of observational studies

Item	No.	Recommendation	Page / Lines	Description of compliance
**TITLE AND ABSTRACT**				
**Title and abstract**	1a	(a) Indicate the study's design with a commonly used term in the title or the abstract	Title; Abstract Methods<br>L. 16 –20	The title explicitly states "Network Psychometric Analysis" and "Lebanese adults". The Methods heading in the structured abstract states "A cross-sectional convenience-sampled online survey among 406 Lebanese adults."
	1b	(b) Provide an informative and balanced summary of what was done and what was found	Abstract (all headings)<br>L. 14 – 33	This study aimed to identify independent predictors of DDRK and cardiovascular health (CVH) literacy among Lebanese adults using a psychometric framework. We found that DDRK is closely linked to Mediterranean diet adherence and CV health literacy. Health programs must prioritize the central DDRK hub to make lifestyle intervention more effective.<br>Results include specific statistics: rs, aOR, 95% CI, p-values for all key findings.
**INTRODUCTION**				
**Background / rationale**	2	Explain the scientific background and rationale for the investigation being reported	Introduction; L. 39–113	Introduction establishes the evidence base linking DDRK to lifestyle behaviours, reviews MENA-region and Lebanese-specific nutrition knowledge literature, and identifies the gap: no prior study has applied the GNKQ DDRK subscale alongside MEDAS, GPAQ, and cardiovascular biomarker awareness in Lebanon.
**Objectives**	3	State specific objectives, including any prespecified hypotheses	L. 108–113	The present study aimed to examine the structural relationships between lifestyle factors, DDRK, and cardiovascular health metrics among Lebanese adults. Specifically, we sought to: (a) identify the independent

				lifestyle and sociodemographic predictors of DDRK; (b) determine the behavioral predictors of self-reported cardiovascular health literacy and self-reported biomarker awareness; and (c) characterize the system-level connectedness of these domains using network psychometric analysis.
**METHODS**				
**Study design**	4	Present key elements of study design early in the paper	Section 2.2; L. 126–145; Figure 1	Cross-sectional convenience-sampled online survey. Design described at the opening of Section 2.2. CONSORT-style flow diagram (Figure 1) illustrates participant selection process. STROBE compliance noted in Section 2.2., [32].
**Setting**	5	Describe the setting, locations, and relevant dates, including periods of recruitment, exposure, follow-up, and data collection	Section 2.2; L. 150–152	English online survey disseminated via social media platform across Lebanon. A pilot study conducted with 22 participants prior to full launch. Data collection: May–August 2023.
**Participants**	6a	(a) Give the eligibility criteria, and the sources and methods of selection of participants	Section 2.2; L. 126–138; Figure 1	Inclusion criteria: informed consent, aged 20–65 years, non-pregnant, non-athlete, Lebanese resident, absence of current medical condition, no weight-affecting medications. Exclusion criteria and exclusion counts detailed in Figure 1 (622 accessed → 216 excluded → 406 included).
	6b	(b) For matched studies, give matching criteria and number of exposed and unexposed; for cross-sectional studies, describe how participants were identified	N/A	Not applicable. Cross-sectional study; no matching performed.

**Variables**	7	Clearly define all outcomes, exposures, predictors, potential confounders, and effect modifiers	Sections 2.3.1–2.3.6 L. 154–235	Primary outcome: continuous DDRK score (0–21; GNKQ Section 4). Secondary outcome: binary cardiovascular biomarker unawareness (coded 0/1). CVH literacy score (0–5; continuous). Predictors: MEDAS score (0–14), MET-min/week (continuous), BMI (kg/m^2; continuous), smoking status (3-level), age (continuous). Confounders: sex, education, income (screened via bivariate analyses in Section 2.4.1). Sensitivity analyses: (1) continuous CVH literacy score as dependent variable via multivariable linear regression (Table S3); (2) nonparanormal transformation applied prior to network analysis (Table S5).
**Data sources / measurement**	8	For each variable of interest, give sources of data and details of methods of assessment	Sections 2.3.1–2.3.6; L. 154–235 Table S1	DDRK: GNKQ Section 4, 16 items, score 0–21 [44], validated for Arab population [43]. Mediterranean diet adherence: MEDAS 14-item screener [35,39], previously utilized as standard proxy in Lebanon [33,36–38]. Physical activity: GPAQ 16 items, WHO [40], widely used in the Middle East [41]. BMI: self-reported weight/height drop-down. CVH literacy: predefined clinical range categories for 5 biomarkers based on NCEP ATP III [45] and ADA [46] guidelines; "I don't know" option provided; 3-month recall timeframe specified (L. 212–213). Composite variables derived in Excel; formula in Table S1.
**Bias**	9	Describe any efforts to address potential sources of bias	Section 2.3.6 L. 212–213 Section 4 Limitations L. 636–668	Social desirability bias minimised by providing an explicit "I don't know" response option for all biomarker items. Biomarker recall timeframe specified as within the preceding 3 months (Section 2.3.6, L. 212–213). Selection bias from convenience sampling acknowledged; online platform over-represents educated urban participants and underrepresents rural and economically disadvantaged

				areas. Recall bias for biomarker values addressed by using predefined clinical range categories rather than free numerical recall. Conservative underestimation of unawareness acknowledged as a result of self-report design.
**Study size**	10	Explain how the study size was arrived at	Table 5; Table 6; Table S2; Table S3; Section 4 Strengths L. 672–680	Non-probability convenience sample; no a priori sample size calculation was performed. A post-hoc sensitivity power analysis was run to confirm the adequacy of our sample size relative to the primary outcome(unaware verus Aware). Three converging post-hoc indicators confirm the statistical adequacy of N = 406. (1) Both regression models reached high significance: MLR $F(7,398) = 9.279$, $p < 0.001$ (Table 5); logistic regression $\Delta\chi^2(9) = 49.065$, $p < 0.001$ (Table 6). (2) EPV ratio = 29.6 (266 unaware / 9 predictors; Table S2 / Table 6), exceeding recommended EPV ≥ 10 [81,82]. (3) Observations-to-node ratio = 67.7 (406 / 6 nodes), exceeding the recommended minimum of 3–5 per variable [47]. Sensitivity analysis using continuous CVH literacy score confirmed robustness (Table S3; $F(9,396) = 5.183$, $p < 0.001$).
**Quantitative variables**	11	Explain how quantitative variables were handled in the analyses; describe groupings if applicable	Section 2.4.1–2.4.2; Table S3 Table 3 Table 6	DDRK score retained as continuous (0–21) throughout all multivariable models; no dichotomisation applied. MEDAS score and MET-min/week entered as continuous predictors in regression models. BMI entered as continuous (kg/m^2). CVH literacy score used as continuous node in network analysis and as continuous dependent variable in sensitivity regression (Table S3); used as binary outcome (cardiovascular biomarker unawareness) in primary logistic regression (Table 6). Categorical classifications (PA level; AMD; BMI

				category) used for descriptive and bivariate analyses only (Table 3).
**Statistical methods**	12a	(a) Describe all statistical methods, including those used to control for confounding	Section 2.4.1–2.4.4<br>L. 238-284	Bivariate: Welch's t-test (sex), Welch's one-way ANOVA (all multi-group categorical predictors); Spearman rank-order correlations (continuous predictors). Multivariable linear regression (Enter method, 7 predictors) predicting DDRK. Multivariable binary logistic regression (Enter method, 9 predictors) predicting cardiovascular biomarker unawareness. Parallel multivariable linear regression using continuous CVH literacy score (0–5) as sensitivity analysis (Table S3). Regularised GGM network (EBICglasso, $\gamma = 0.5$); nonparanormal transformation applied prior to network estimation to account for non-normal and bimodal distribution of CVH literacy variable. All analyses conducted in JASP v0.97 (Intel). $\alpha = 0.05$. The data registry can be accessed in OSF directly at https://osf.io/57unh/overview?view_only=fc51b78c45eb49e1ad31519d60a8c753.
	12b	(b) Describe any methods used to examine subgroups and interactions	N/A	No formal subgroup or interaction analyses performed. DDRK quartile and AMD subgroup comparisons of CVD health literacy score conducted as descriptive analyses only (Figure 2C, Supplementary Table S2); no inferential testing by subgroup interaction.
	12c	(c) Explain how missing data were addressed	Section 2.2; Figure 1	No missing data in the analytical dataset. Incomplete questionnaires were excluded prior to analysis at the data collection stage (n = 0 missing for any analytical variable). Income skip-logic was handled structurally: non-employed participants were routed past the income question and classified as

				"Not employed/No income" (n = 87); no imputation required.
	12d	(d) Describe how loss to follow-up was addressed	N/A	Not applicable. Cross-sectional study with single time-point data collection; no follow-up period.
	12e	(e) Describe any sensitivity analyses	Section 2.4.2<br>Section 2.4.3<br>L. 267–269<br>L. 275–277<br>Table S3<br>Table S5	Two sensitivity analyses were performed. (1) A parallel multivariable linear regression was conducted using the continuous CVH literacy score (0–5) as the dependent variable (instead of the binary unawareness variable) to confirm the robustness of the primary logistic regression model findings (Table S3). (2) A nonparanormal transformation was applied to the dataset prior to EBICglasso network analysis to ensure robustness against the non-normal and bimodal distribution of the CVH literacy variable; stable parameter matrices confirmed (Table S5).
**RESULTS**				
**Participants**	13a	(a) Report numbers of individuals at each stage of study	Section 2.2; Figure 1<br>L. 129–134	622 participants accessed the survey; 216 excluded (reasons: refusal n=2, age ≤19 n=39, age >65 n=71, pregnancy n=16, non-Lebanese residency n=26, athlete n=44, medical conditions/medications n=18); 406 included in final analysis.
	13b	(b) Give reasons for non-participation at each stage	Figure 1<br>L. 130-134	All exclusion reasons with counts provided in the CONSORT flow diagram (Figure 1) and listed in Section 2.2.
	13c	(c) Consider use of a flow diagram	Figure 1<br>L. 141-144	CONSORT-style flow diagram provided (Figure 1).
**Descriptive data**	14a	(a) Give characteristics of study participants and information on exposures and	Section 3.1; Table 1<br>L. 291–306	Table 1 presents full sociodemographic, anthropometric, lifestyle, dietary, and physical activity characteristics of all 406 participants. Mean DDRK score, MEDAS score,

		potential confounders		BMI, age, and MET-min/week reported with SD.
	14b	(b) Indicate number of participants with missing data for each variable of interest	Section 2.2; Figure 1	No missing data in any analytical variable. Stated explicitly in Section 2.2 and confirmed in Figure 1.
	14c	(c) Summarise follow-up time	N/A	Not applicable. Cross-sectional single time-point design.
**Outcome data**	15	Cross-sectional study: report numbers of outcome events or summary measures	Section 3.5; Table 1; Supplementary Table S2	DDRK score distribution: mean 12.5 ± 4.0, range 0–21. CVD biomarker unawareness: 266/406 (65.5%) classified as lacking awareness. CVD health literacy score distribution reported in Supplementary Table S2 and Figure 2B.
**Main results**	16a	(a) Give unadjusted and adjusted estimates and their precision (95% CI)	Table 4, L.330<br>Table 5, L.341<br>Table 6, L.394	Table 4: Spearman correlations with rs and p-values. Table 5: MLR unstandardised (B) and standardised (β) coefficients, 95% CI, p-values, VIF. Table 6: logistic regression aOR, 95% CI, p-values. Model fit statistics (R^2, F, Nagelkerke R^2, $\Delta\chi^2$, Hosmer-Lemeshow) reported in table model summary rows.
	16b	(b) Report category boundaries when continuous variables were categorised	Table 3; L.178-195; Section 2.3.3; 2.3.4,	MEDAS categories: Low 0–5, Fair 6–9, High 10–14 (Schröder et al., 2011). PA categories: Low <600, Moderate >1,500, High >3,000 MET-min/week (WHO 2020). BMI categories: <18.5 (underweight), 18.5–24.9 (normal), 25.0–29.9 (overweight), ≥30 (obese). DDRK quartile boundaries reported in Figure 2C and Supplementary Table S2.
	16c	(c) If relevant, consider translating estimates of relative risk into absolute risk	Section 3.6 L.399-407	Odds reduction per unit interpreted as percentage change: DDRK aOR = 0.878 → 12.2% lower odds per point; MEDAS aOR = 0.842 → 15.8% lower odds per point; Age aOR = 0.961 → 3.9% lower odds per year.

**Other analyses**	17	Report other analyses done — eg subgroups, interactions, sensitivity analyses	Section 2.4.2; Section 2.4.3; Figure 2C; Table S2; Table S3; Table S5; Figure 3	CVH literacy score gradient across DDRK quartiles and AMD levels (Figure 2C; Table S2) presented as descriptive subgroup analysis. Sensitivity analysis 1: parallel multivariable linear regression using continuous CVH literacy score as dependent variable (Table S3). Sensitivity analysis 2: nonparanormal transformation applied prior to EBICglasso network estimation to account for bimodal CVH literacy distribution; stable parameters confirmed (Table S5). Network psychometric analysis as primary integrative analysis (Section 2.4.3; Section 3.7; Figure 3).
**DISCUSSION**				
**Key results**	18	Summarise key results with reference to study objectives	Section 4; L. 454–467	Discussion opens by addressing each of the three stated objectives: (a) MEDAS, smoking, PA, BMI as DDRK predictors; (b) DDRK, MEDAS, age as protective against biomarker unawareness; (c) DDRK as central network hub. The CVH literacy education paradox is explicitly stated and contextualized within the Lebanese healthcare landscape.
**Limitations**	19	Discuss limitations of the study, taking into account sources of potential bias or imprecision	Limitations section; L. 636–668	Limitations addressed: (1) cross-sectional design precludes causal inference; (2) self-reported biomarker values (recall bias, potential misclassification); 3-month recall timeframe specified to reduce ambiguity; (3) self-reported height/weight for BMI; (4) online convenience sampling (selection bias, internet-access requirement, overrepresentation of Mount Lebanon and educated participants; underrepresentation of rural and economically disadvantaged areas); (5) conservative underestimation of unawareness prevalence due to social

				desirability bias. Directionality of bias discussed for each limitation.
**Interpretation**	20	Give a cautious overall interpretation of results considering objectives, limitations, multiplicity of analyses, results from similar studies, and other evidence	Section 3; Section 4; L. 488–631	Results framed as observational associations throughout. Causal inference explicitly avoided. Network centrality findings explicitly described as exploratory and hypothesis-generating (L. 422–424; L. 591–595; L. 705–711). Findings contextualized within Lebanese and global literature on nutrition knowledge, Mediterranean diet adherence, and cardiovascular health literacy. The "knowledge-behaviour gap" concept invoked to explain discordance between high education and poor biomarker awareness [52].
**Generalisability**	21	Discuss the generalisability (external validity) of the study results	Limitations; Section 4; L. 644–648; L. 522–526	Generalisability acknowledged as limited: convenience sample recruited online, overrepresenting Mount Lebanon (62.8%), educated (84% university-level), and single participants (56.7%). Results may not fully represent rural or economically disadvantaged areas of Lebanon where healthcare access is even more limited, particularly regions affected by the economic crisis (South Lebanon, Beqaa, North Lebanon). Online sampling also likely underrepresents individuals with the lowest digital literacy and least access to healthcare.
**OTHER INFORMATION**				
**Funding**	22	Give the source of funding and the role of the funders for the present study	Funding statement	No external funding received. Stated explicitly in the Funding statement: "This research received no external funding." No role of funder to declare.

Note: An Explanation and Elaboration article discusses each checklist item and gives methodological background and published examples of transparent reporting. The STROBE checklist is best used in conjunction with this article (freely available on the STROBE website at www.strobe-statement.org). STROBE = STrengthening the Reporting of OBservational Studies in Epidemiology.
