# Supplementary material for: Lifestyle Factors and Diet-Disease-Related Knowledge: A Network Psychometric Analysis of Cardiovascular Health Literacy Among Lebanese Adults
Source: Nutrients. 2026 Jul 6;18(13):2196. doi: 10.3390/nu18132196 (PMC13364409; doi:10.3390/nu18132196)
Supplement: Supplementary file 1 [file nutrients-18-02196-s001.zip › nutrients-4396486-tables.pdf]

Table S1. Operational Definitions and Scoring Formulas for Cardiovascular Health Variables

Variable	Source columns	Scoring rule	Excel formula
**CV biomarker unawareness**<br>*(Binary: 0/1)*	T2: Total Cholesterol<br>U2: LDL<br>V2: HDL<br>W2: Triglycerides<br>X2: HbA1c	Coded as 1 if the participant selected "I don't know" for **any** of the five cardiovascular biomarkers; otherwise coded as 0.	=IF(OR(T2="I don't know", U2="I don't know", V2="I don't know", W2="I don't know", X2="I don't know"), 1, 0)
**CV health Literacy Score**<br>*(Continuous: 0–5)*	T2: Total Cholesterol<br>U2: LDL<br>V2: HDL<br>W2: Triglycerides<br>X2: HbA1c	Sum of all known cardiovascular biomarkers, yielding a cumulative spectrum score from 0 (none known) to 5 (all known).	=COUNTIF(T2:X2, "<>I don't know")

CV: cardiovascular

Table S2. Cardiovascular biomarker awareness and cardiovascular health literacy score profile of the study sample (n = 406)

Variable	n	CV biomarker unawareness[1] % (n)	CV biomarker awareness[1] % (n)	CV health literacy score[2] Mean ± SE
Total cholesterol (tCHOL)	406	55.7 (226)	44.3 (180)	
LDL	406	58.6 (238)	41.4 (168)	
HbA1c	406	61.6 (250)	38.4 (156)	
HDL	406	59.1 (240)	40.9 (166)	
Triglycerides (TG)	406	57.9 (235)	42.1 (171)	
Composite[3]	406	65.5 (266)	34.5 (140)	
Score 0 (no biomarker known)	217	53.4		
Score 1	11	2.7		
Score 2	7	1.7		
Score 3	8	2		
Score 4	23	5.7		
Score 5 (all biomarkers known)	140	34.5		
DDRK knowledge quartile (KW = 18.549, $p < 0.001$, $\varepsilon^2 = 0.039$)				
Q1 (DDRK score ≤ 10)	127			1.43 ± 0.19
Q2 (DDRK score 11–13)	103			2.21 ± 0.23
Q3 (DDRK score 14–15)	81			2 ± 0.26
Q4 (DDRK score ≥ 16)	95			2.83 ± 0.24
AMD level (KW = 19.641, $p < 0.001$, $\varepsilon^2 = 0.044$)				
Low	93			1.51 ± 0.22
Fair	256			2.03 ± 0.15
High	57			3.19 ± 0.31

[1] Unaware = responded "I don't know"; Aware = selected any predefined clinical range value. Participants were presented with clinical range categories and asked to identify the range corresponding to their most recent laboratory results.

[2] CVD health literacy score (0–5): computed by summing the five cardiovascular biomarkers (tCHOL, LDL, HDL, TG, HbA1c) for which participants selected a known clinical range value.

[3] The composite unawareness captures participants who have at least one biomarker unknown (score ≤ 4) of tCHOL, LDL, HbA1c, TG, or HDL. Conversely, composite awareness corresponds to a score of exactly 5, with all five markers known. Participants scoring 5 were classified as fully aware.

Abbreviations: CV, Cardiovascular; DDRK, diet-disease-related knowledge; AMD, adherence to the Mediterranean diet; HbA1c, glycated haemoglobin; LDL, low-density lipoprotein; HDL, high-density lipoprotein; TG, triglycerides; SE, standard error of the mean; KW, Kruskal-Wallis test statistic; ε^2,epsilon-squared effect size.

Table S3. Multivariate linear Regression predicting cardiovascular health literacy score (n = 406)

Predictor Variable	B	SE	β	95% CI	p-value
**Model Summary: $R^2 = 0.105$ | Adjusted $R^2 = 0.085$ | $F(9, 396) = 5.183$, $p < 0.001$**					
Intercept	-1.781	0.981	—	[-3.709, 0.148]	0.07
DDRK score	0.134	0.03	0.229	[0.075, 0.193]	< .001
MEDAS score	0.119	0.055	0.108	[0.010, 0.227]	0.032
Age	0.038	0.011	0.166	[0.015, 0.060]	0.001
PA (MET-min/week)	0.0001	0.0001	0.042	[-0.000, 0.000]	0.406
BMI (kg/m^2)	0.007	0.029	0.013	[-0.049, 0.064]	0.8
Education level [b]					
Postgraduate	0.561	0.294	—	[-0.016, 1.139]	0.057
Undergraduate	-0.042	0.315	—	[-0.658, 0.574]	0.893
Smoking status [c]					
Never smoker	-0.452	0.254	—	[-0.952, 0.047]	0.076
Ex-Smoker	-0.072	0.465	—	[-0.986, 0.843]	0.878

[a] Reference category: Female.

[b] Reference category: Graduate education.

[c] Reference category: Current smoker

B, Unstandardised coefficient; SE, Standard error; aOR, adjusted odds ratio; CI, confidence interval; DDRK, diet-disease-related knowledge; MEDAS, Mediterranean diet adherence screener; BMI, body mass index; MET-min/week, metabolic equivalent minutes per week; PA, physical activity.

Table S4: Global network architecture and sparsity parameters

Number of nodes	Number of non-zero edges	Sparsity
6	13 / 15	0.133

Table S5. Sensitivity Analysis Network Parameters: Nonparanormal Regularized Partial Correlation Weights Matrix and Standarized Node Centrality Indicators

Variable	DDRK	BMI[a]	Age	PA[b]	MEDAS	CVH lit	Betweenness	Closeness	Strength	Exp. Influence	Clustering
DDRK	0	-0.128	0	0.119	0.185	0.156	0.5	1	1	0.805	1.06
BMI[a]	-0.128	0	0.197	-0.111	-0.066	0	0.5	0.858	0.855	-0.26	-0.824
Age	0	0.197	0	-0.182	0	0.133	1	0.874	0.872	0.36	-0.262
PA[b]	0.119	-0.111	-0.182	0	0.096	0.032	0.5	0.883	0.92	-0.11	-0.359
MEDAS	0.185	-0.066	0	0.096	0	0.091	0	0.733	0.746	0.745	1.402
CVH lit	0.156	0	0.133	0.032	0.091	0	0	0.809	0.701	1	-1.017

DDRK, Diet-Disease-Related Knowledge); MEDAS, Mediterranean Diet Adherence Screener; BMI, Body mass index; PA, physical activity; CVH literacy, cardiovascular health literacy.

[a] BMI expressed in kg/m^2

[b] PA expressed in MET-min/week (Metabolic equivalent minutes per week).

Table S6: Regularized partial correlation edge weights matrix across the 6 network nodes

Variable	Network					
	DDRK	BMI	Age	PA	MEDAS	CVH literacy
DDRK	0.000	-0.125	-0.040	0.102	0.172	0.202
BMI[a]	-0.125	0.000	0.193	-0.106	-0.096	0.000
Age	-0.040	0.193	0.000	-0.167	0.000	0.168
PA[b]	0.102	-0.106	-0.167	0.000	0.051	0.036
MEDAS	0.172	-0.096	0.000	0.051	0.000	0.114
CVH literacy	0.202	0.000	0.168	0.036	0.114	0.000

DDRK, Diet-Disease-Related Knowledge); MEDAS, Mediterranean Diet Adherence Screener; BMI, Body mass index; PA, physical activity; CVH literacy, cardiovascular health literacy.

[a] BMI expressed in kg/m^2

[b] PA expressed in MET-min/week (Metabolic equivalent minutes per week).

Table S7: Centrality measures per variable

Variable	Network			
	Betweenness	Closeness	Strength	Expected influence
Age	1.000	0.923	0.885	0.297
BMI[a]	0.000	0.894	0.811	-0.257
CVH literacy	1.000	0.923	0.812	1.000
DDRK	0.500	1.000	1.000	0.598
MEDAS	0.000	0.713	0.677	0.464
PA[b]	0.000	0.746	0.720	-0.159

DDRK, Diet-Disease-Related Knowledge); MEDAS, Mediterranean Diet Adherence Screener; BMI, Body mass index; PA, physical activity assessed by MET-min/week (Metabolic equivalent minutes per week); CVH literacy, cardiovascular health literacy.

[a] BMI expressed in kg/m^2

[b] PA expressed in MET-min/week (Metabolic equivalent minutes per week)

Table S8: Local network clustering coefficients per variable

Variable	Network			
	Barrat	Onnela	WS	Zhang
DDRK	-1.017	0.086	-1.291	-0.571
CVH literacy	0.532	-0.160	0.645	-0.520
MEDAS	1.527	-0.081	0.645	1.340
PA[a]	-0.217	-1.736	-1.291	1.109
Age	-1.106	0.660	0.645	-1.157
BMI[b]	0.281	1.229	0.645	-0.201

DDRK, Diet-Disease-Related Knowledge); MEDAS, Mediterranean Diet Adherence Screener; BMI, Body mass index; PA, physical activity; CVH literacy, cardiovascular health literacy.

[a] PA expressed in MET-min/week (Metabolic equivalent minutes per week).

[b] BMI expressed in kg/m^2
